# Spiculation Sign Recognition in a Pulmonary Nodule Based on Spiking Neural P Systems

**DOI:** 10.1155/2020/6619076

**Published:** 2020-12-23

**Authors:** Shi Qiu, Jingtao Sun, Tao Zhou, Guilong Gao, Zhenan He, Ting Liang

**Affiliations:** ^1^Key Laboratory of Spectral Imaging Technology CAS, Xi'an Institute of Optics and Precision Mechanics, Chinese Academy of Sciences, Xi'an 710119, China; ^2^Department of Radiology, The First Affiliated Hospital of Xi'an Jiaotong University, Xi'an 710061, China; ^3^School of Computer Science and Engineering, North Minzu University, Yinchuan 750021, China; ^4^School of Science, Ningxia Medical University, Yinchuan 750004, China; ^5^Key Laboratory of Ultra-Fast Photoelectric, Xi'an Institute of Optics and Precision Mechanics, Chinese Academy of Sciences Xi'an, 710119, China; ^6^Shaanxi Institute of Medical Device Quality Supervision and Inspection, Xi'an 712046, China; ^7^Department of Biomedical Engineering, the Key Laboratory of Biomedical Information Engineering of the Ministry of Education, School of Life Science and Technology, Xi'an Jiaotong University, Xi'an 710061, China

## Abstract

The spiculation sign is one of the main signs to distinguish benign and malignant pulmonary nodules. In order to effectively extract the image feature of a pulmonary nodule for the spiculation sign distinguishment, a new spiculation sign recognition model is proposed based on the doctors' diagnosis process of pulmonary nodules. A maximum density projection model is established to fuse the local three-dimensional information into the two-dimensional image. The complete boundary of a pulmonary nodule is extracted by the improved Snake model, which can take full advantage of the parallel calculation of the Spike Neural P Systems to build a new neural network structure. In this paper, our experiments show that the proposed algorithm can accurately extract the boundary of a pulmonary nodule and effectively improve the recognition rate of the spiculation sign.

## 1. Introduction

A pulmonary nodule is an early pattern of lung cancer. Malignant lesions might occur in some pulmonary nodules and even threaten patients' lives seriously [1]. The spiculation sign is the feature of a pulmonary nodule. It is a radial and unbranched strip shadow extending from the boundary of a pulmonary nodule to the surrounding pulmonary parenchyma [2]. Its detection may cost more time and energy of doctors.

The diagnosis of benign and malignant pulmonary nodules can be divided into imaging detection and “biopsy.” The most accurate detection method is “biopsy,” but it cannot predict the development trend of pulmonary nodules. Imaging analysis is still the mainstream detection method [3, 4]. It is also a main method to predict the development trend of benign and malignant pulmonary nodules from the perspective of imaging [5, 6]. “Biopsy” needs to sample the suspected lung lesions for detection. In the detection process, the instrument needs to be deep into the lung, which is easy to cause discomfort to patients. The suspected area for “biopsy” should be determined in advance. It needs to be analyzed by modeling from the perspective of imaging, so it is very important to start prepositioning from the perspective of imaging. “Biopsy” is the gold standard for judging benign and malignant pulmonary nodules. But the probability of malignant pulmonary nodules is far less than that of malignant. For this reason, not all pulmonary nodules must be biopsied. Main imaging features of pulmonary nodules include lobulation sign, spiculation sign, and cavity sign. It is necessary to identify the signs in biopsy of high probability pulmonary nodules. According to the sign features of pulmonary nodules, a single model cannot realize recognition accurately and effectively. Therefore, we need to analyze the signs and establish the model one by one. “Biopsy” can only detect the current benign and malignant pulmonary nodules, but cannot predict the development of pulmonary nodules. But the imaging is different, it can compare the change rate of the same lesion point in different time periods, predict the development area of pulmonary nodules in the future, and further guide the diagnosis. Therefore, our research is significant.

The main signs of pulmonary nodules are lobulation sign, spiculation sign, cavity sign, and calcification. The spiculation sign has the highest deterioration rate, and it is difficult to distinguish the lobulated sign. Therefore, our research is based on the spiculation sign in this paper. Pulmonary nodules present a limited number of pixels in the image, and pulmonary nodules are volume data with three-dimensional structure. As a result, CT cannot accurately locate the signs of pulmonary nodules and make accurate judgment. Aiming at this problem, a density projection algorithm is proposed to integrate local 3D information into two-dimensional images for accurate diagnosis.

With the development of computer imaging technology, computer-aided diagnosis becomes possible for doctors and also has been successfully applied into the detection of pulmonary nodules: Qiu et al. [2] establishes a model to detect solitary pulmonary nodules. Gavrielides et al. [7] built a three-dimensional model to analyze the volume of pulmonary nodules. El-Baz et al. [8] judges the malignant degree of pulmonary nodules through analyzing morphological characteristics of pulmonary nodules. Brandman and Ko [9] establish a complete process including the detection of pulmonary nodules and the distinguishment and management of signs. Chen et al. [10] establish a neural network and a regression model to distinguish pulmonary nodules. Huang et al. [11] introduce the practical application of membrane calculation and achieves good results. Fan et al. [12] analyze the sign of pulmonary nodules from a mathematical and statistical perspective. Vinay et al. [13] construct an optimal classifier to distinguish the spiculation sign from a three-dimensional perspective. Dhara et al. [14] quantify the speculation sign on the basis of a three-dimensional model. Han et al. [15] focus on boundary characteristics to analyze the benign and malignant pulmonary nodules. Wang et al. [16] establish an image enhancement model to highlight pulmonary nodules. Choi and Choi [17] use a fixed threshold to segment pulmonary nodules. Rubin [18] sets seed points for local growth of pulmonary nodules. Shen et al. [19] establish a bidirectional coding system to improve the efficiency of the proposed algorithm. Qiang et al. [20] apply the active contour model for the segmentation of pulmonary nodules. Messay et al. [21] realize the segmentation of pulmonary nodules through analyzing the characteristics of CT pixel distribution from the linear regression perspective. Zhang et al. [22] analyze the spiking neural P systems based on the principle and puts forward a fast solution algorithm. Kumar et al. [23] classify pulmonary nodules by depth features. Bartholmai et al. [24] analyze the characteristics of pulmonary nodules with a computer. Firmino et al. [25] analyze the malignant degree of pulmonary nodules from the sign perspective. Dhara et al. [26] establish a gradient model to extract pulmonary nodules. Gonçalves et al. [27] establish the Hessian matrix to segment pulmonary nodules. Wang et al. [28] establish a data-driven model to focus on the pulmonary nodule area. Soliman et al. [29] establish the Adaptive Appearance-Guided Shape Model to simulate the distribution of pulmonary nodules. Froz et al. [30] classify pulmonary nodules with the support vector machine. Hoogi et al. [31] improve the level set algorithm for the pulmonary nodule segmentation. Wang et al. [32] apply the spiking neural P systems to realize the target tracking and path planning. Shakir et al. [33] establish a three-dimensional level set algorithm based on the two-dimensional segmentation. Qiu et al. [34] classify pulmonary nodules based on the geometric theory. Xie et al. [35] fuse multiple features to distinguish pulmonary nodules. Wang et al. [36] propose a set of complete data training algorithm to classify pulmonary nodules. Pang et al. [37] Automatic lung segmentation based on texture and deep features of hrct images with interstitial lung disease. Rong et al. [38] improve the spike neural P systems and improve the diagnosis accuracy. Cao et al. [39] used two-stage convolutional neural networks for nodule detection. Xu et al. [40] used multiresolution CT screening images to detect nodules.

Currently, the main problems of the computer-aided diagnosis of pulmonary nodules can be summarized as follows: (1) the two-dimensional and three-dimensional features of pulmonary nodules are difficult to be balanced during the modeling process. (2) The accurate segmentation of pulmonary nodules cannot be realized with gray values and without boundary features. (3) An effective distinguishing mechanism cannot be established after obtaining features of pulmonary nodules.

Therefore, in this paper, a spiculation sign recognition algorithm is proposed after studying the doctors' diagnosis process of pulmonary nodules. (1) A maximum intensity projection model is established to fuse the three-dimensional information into the two-dimensional image to reduce the missed rate of spiculation signs. (2) The accurate extraction of pulmonary nodules can be realized by the improved Snake model to strengthen the boundary effect. (3) A neural network framework based on the Spike Neural P Systems is constructed through focusing on boundary features of pulmonary nodules.

## 2. Algorithm

**Figure 1:**
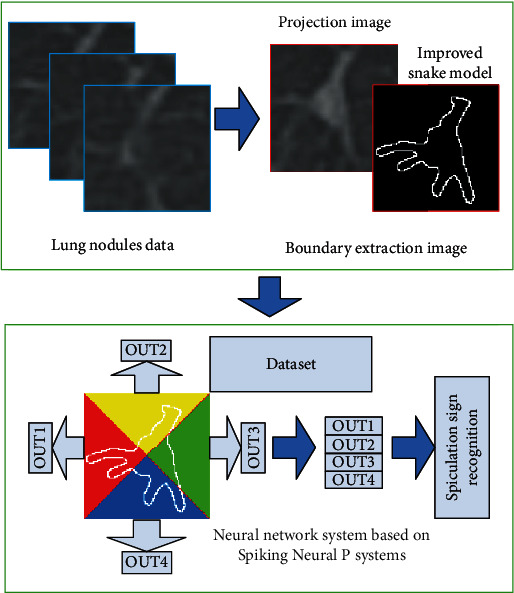
Flow chart of the spiculation sign recognition algorithm.

The spiculation sign recognition process of pulmonary nodules is simulated by the computer, as shown in Figure 1. (1) The maximum intensity projection algorithm is constructed to fully display the features of pulmonary nodules. (2) The boundary of pulmonary nodules is focused by the improved Snake algorithm. (3) The Spiking Neural P Systems is optimized to realize the sign recognition.

### 2.1. Projection Algorithm

The spiculation sign is the main feature to distinguish benign pulmonary nodules from malignant ones. It is defined as a radial and unbranched stripe shadow extending from the boundary of a pulmonary nodule to the surrounding pulmonary parenchyma. According to the local highlight of a pulmonary nodule, its section structure is extracted layer by layer to construct a model from the perspective of local three-dimensional information.

**Figure 2:**
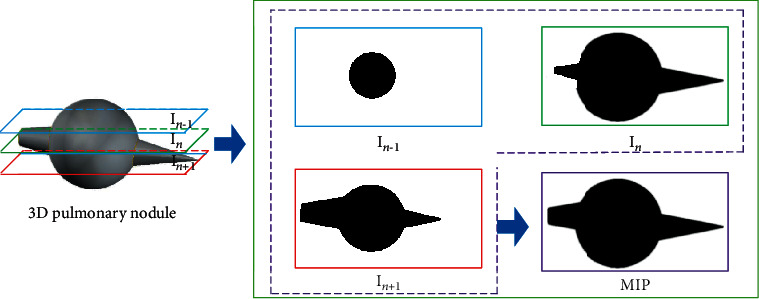
MIP effect image.

The maximum gray value along the ray direction of continuous multiframes is used by MIP as the gray value of the corresponding point on the projection image [41],

(1) $$\begin{array}{c} \text{MIP}\left(x,y\right)=\max\left(I_{0}\left(x,y\right)\cdots I_{N}\left(x,y\right)\right), \end{array}$$

where MIP(*x*, *y*) is the gray value at the point (*x*, *y*) on the MIP image. *N* is the number of projection layers. *I*_*k*_(*x*, *y*) is the gray value at the point (*x*, *y*) on the *k*-th image in the original CT sequence images. MIP images contain local three-dimensional features, which can restore the local three-dimensional information of pulmonary nodules, as shown in Figure 2.

### 2.2. The Segmentation Algorithm of Pulmonary Nodule

A pulmonary nodule is displayed in the highlighted area and occupies a limited number of pixels in CT images.

A benign pulmonary nodule has features of small area, high luminance, and smooth boundary; however, a malignant pulmonary nodule has features of large area, high luminance, and blurred boundary. Complete segmentation is the premise of the pulmonary nodule distinguishment.

#### 2.2.1. The Snake Model

The Snake model algorithm can perform the target segmentation from the perspective of internal energy and external energy [42]. It has the following advantages: image data, initial estimation, target contour, and knowledge-based constraints are unified in one process. It can automatically converge to the state of minimum energy after proper initialization. Minimizing the energy from coarse to fine in scale space can greatly expand the capture area and reduce the complexity. Meanwhile, the Snake model algorithm also has its disadvantages: It is sensitive to the initial position, and Snake needs to be placed near the image features depending on other mechanisms. It may converge to the local extremum or even diverge because of the nonconvexity of the Snake model. Dong et al. [43] introduce the deep learning theory to constrain the Snake algorithm to segment targets. Rajinikanth et al. [44] achieve the three-dimensional target segmentation based on the Snake algorithm and the Otsu algorithm. Ma et al. [45] fuse the local phase position, and the Snake algorithm alleviates the problem of convergence to the local extremum.

When the Snake model achieves the balance of internal energy and external energy, the optimal segmentation effect is obtained. The energy functional is defined as:

(2) $$\begin{array}{c} \left\{\begin{array}{c} E=\int_{0}^{1}\left\{E_{\text{in}}\left[C\left(s\right)\right]+\right.\left.E_{\text{out}}\left[C\left(s\right)\right]\right\}ds,\\ C\left(s\right)=\left(x\left(s\right),y\left(s\right)\right)s\in \left[0,1\right], \end{array}\right. \end{array}$$

where *C*(*s*) is a contour curve and *E*_in_[*C*(*s*)] is an internal energy function. *E*_in_[*C*(*s*)] is only related to the curve itself, so that the curve keeps continuity and smoothness during deformation. *E*_out_[*C*(*s*)] is an external energy function, and *E*_out_[*C*(*s*)] is only related to the image itself, which can drive the curve to move towards the target boundary continuously.

(3) $$\begin{array}{c} E_{\text{in}}\left[C\left(s\right)\right]=\frac{1}{2}\left[\alpha {\left|C^{\prime }\left(s\right)\right|}^{2}+\beta {\left|C^{\prime \prime }\left(s\right)\right|}^{2}\right], \end{array}$$

where *α* is the elastic energy weight coefficient and *β* is the rigid energy weight. The minimization of variational principle *C*(*s*) should satisfy the Euler equation:

(4) $$\begin{array}{c} \alpha C^{\prime \prime }-\beta {C^{\prime \prime }}^{\prime \prime }-\nabla E_{\text{out}}=0. \end{array}$$

The GVF model [46] introduces the gradient vector flow *V*(*x*, *y*) = (*u*(*x*, *y*), *v*(*x*, *y*)) to replace the external force of the Snake model, then the energy functional of the external force field is

(5) $$\begin{array}{c} \varepsilon_{\text{GVF}}=\min\left\{\iint \left\{w\left(u_{x}^{2}+u_{y}^{2}+v_{x}^{2}+v_{y}^{2}\right)\right.\right.+\left.\left.{\left|\nabla f\right|}^{2}{\left|V-\nabla f\right|}^{2}\right\}dxdy\right\}, \end{array}$$

where *w* is the weight coefficient to control the smoothness of the external force field. *f*(*x*, *y*) is an image boundary mapping function. When the curve is far from the target contour, the first term plays a major role. On the contrary, the second term plays a major role in expanding the search scope. By solving

(6) $$\begin{array}{c} \left\{\begin{array}{c} w\nabla^{2}u-\left(u-f_{x}\right)\left(f_{x}^{2}+f_{y}^{2}\right)=0,\\ w\nabla^{2}v-\left(v-f_{x}\right)\left(f_{x}^{2}+f_{y}^{2}\right)=0, \end{array}\right. \end{array}$$

where the GVF field is obtained, where▽^2^ is a Laplacian operator. The Laplace operator produces an isotropic smoothing effect on the external force field and cannot protect the boundary.

#### 2.2.2. The Improved Model

As the traditional Snake algorithm is easy to converge to the local extreme and cannot protect boundary, we have analyzed the Laplace operator: The Laplace operator can be decomposed into normal and tangent components, and the normal direction component can promote the contour line to converge to the deep concave part. Thus, *w* | *J*_*v*_*P*∣ term is added to make the curve converge to the small deep concave boundary. The improved function is as follows:

(7) $$\begin{array}{c} \varepsilon =\min\left\{\iint \left\{m\left(x,y\right){\left|\nabla V\right|}^{2}\right.\right.\left.\left.+h\left(x,y\right)\left(w{\left|J_{v}P\right|}^{2}+{\left|V-\nabla f\right|}^{2}\right)\right\}dxdy\right\}, \end{array}$$

where *w*, *g*(*x*, *y*), and *h*(*x*, *y*) are weighting functions and *J*_*v*_ is the Jacobian matrix of external force field. In order to enhance the corresponding boundary, we construct

(8) $$\begin{array}{c} P=\left[\begin{array}{c} -\frac{I_{xy}}{\sqrt{I_{xx}^{2}+I_{xy}^{2}}},\frac{I_{xx}}{\sqrt{I_{xx}^{2}+I_{xy}^{2}}}\\ -\frac{I_{yy}}{\sqrt{I_{yx}^{2}+I_{yy}^{2}}},\frac{I_{yx}}{\sqrt{I_{yx}^{2}+I_{yy}^{2}}} \end{array}\right], \end{array}$$

to increase the accuracy of corner positioning.

In Eq. (7), |▽*V*|^2^ has a strong smoothing effect on the boundary. To reduce boundary weakening, |▽*V*|^2^ is replaced by

(9) $$\begin{array}{c} G={\left(1+{\left|\nabla V\right|}^{2}\right)}^{q\left(\left|\nabla f\right|\right)/2}q\left(\left|\nabla f\right|\right)=1+\frac{1}{1+\left|\nabla f\right|}. \end{array}$$

In the smoothing area, ∣▽*f*  | →0, *q*(∣▽*f* ∣) → 2, the external force field has an isotropic diffusion effect. At the boundary, ∣▽*f* | →∞, *q*(∣▽*f* ∣) → 1, *G* → ∣▽*V*∣, the external force field only diffuses along the boundary direction to prevent boundary leakage and improve the antinoise performance. The energy function is

(10) $$\begin{array}{c} \varepsilon =\min\left\{\iint \left\{m\left(x,y\right)G\right.\right.\left.\left.+h\left(x,y\right)\left(w{\left|J_{v}P\right|}^{2}+{\left|V-\nabla f\right|}^{2}\right)\right\}dxdy\right\}, \end{array}$$

which corresponds to the Euler equations *Z*(*u*) = 0 and *Z*(*v*) = 0.

The iterative formula of numerical solution *l*(*u*_*i*,*j*_^*n*+1^), *l*(*v*_*i*,*j*_^*n*+1^) is

(11) $$\begin{array}{c} l\left(a_{i,j}^{n+1}\right)=\left(1-\Delta t\cdot h\left(\left|\nabla f\right|\right)a_{i,j}^{n}+\Delta t\cdot g\left(\left|\nabla f\right|\right)\right.\left[{\left(1+{\left|\nabla a\right|}^{2}\right)}^{q-2/2}\right.a_{tt}+\left(\left(q-2\right){\left|\nabla a\right|}^{2}{\left(1+{\left|\nabla a\right|}^{2}\right)}^{q-4/2}\right.\left.\left.+{\left(1+{\left|\nabla a\right|}^{2}\right)}^{q-2/2}+1\right)u_{nn}\right]+\Delta t\cdot \left[w^{2}h\left(\left|\nabla f\right|\right)\left(P_{11}^{2}a_{xx}+P_{12}^{2}a_{yy}+2P_{11}P_{12}\right)+c_{a}\right], \end{array}$$

where *c*_*u*_ = *h*(|∇*f*|)*f*_*x*_, *c*_*v*_ = *h*(|∇*f*|)*f*_*y*_. Δ*t* is the iteration step. (*u*_*i*,*j*_^*n*^, *v*_*i*,*j*_^*n*^) represents the field forces at coordinates (*i*, *j*) with *n* iterations. Pulmonary nodules are extracted layer by layer to obtain complete pulmonary nodules.

### 2.3. Neural Network System Based on Spiking Neural P Systems

The SN P systems are a parallel computing model derived from organisms [47]. Wang et al. [48] introduce the fuzzy set theory on the basis of SN P systems, which solves the problem of fault diagnosis to a certain extent. The topological structure of SN P systems is composed of a directed graph. Each neuron in the system is represented by a node, and the synapse between two adjacent neurons in the system is represented by edge. It is similar to the topological structure of the artificial neural network. There are abundant theoretical and applied researches in ANN, so the learning rules in ANN can be introduced into SN P systems.

The neural network based on SN P systems can be defined as

(12) $$\begin{array}{c} \begin{array}{c} \text{B}=\left(O,\sigma_{1},\cdots ,\sigma_{m},\text{syn},y,\text{in},\text{out}\right),\\ \sigma_{i}=\left(n_{i},R_{i}\right), \end{array} \end{array}$$

where *O* represents a set of pulses; *σ*_*m*_ represents the *m*-th neuron in System *B*; *n*_*i*_ ≥ 0 represents the number of original pulses; *R*_*i*_ represents a set of all rules in neuron *σ*_*i*_; The form of excitation rule is *E*/*a*^*c*^ → *a*, *c* ≥ 1; The rule of oblivion is *a*^*s*^ → *λ*, *s* ≥ 1; *y* represents the learning function of system; in and out represent the input and output neurons of the system, respectively. Define the rule as *E*/*a*^*c*^ → *αk*(*i*, *Q*_j_), *k* ≥ 1, *c* ≥ 1, 1 ≤ *j* ≤ ∣*R*_*i*_∣. When the rule is called, all neurons in *σ*_*i*_ and *Q*_*j*_ establish the connection state.

Syn represents a synapse between *σ*_*i*_ and *σ*_*j*_; *w*_*ij*_(*t*) represents the weight of synapse (*i*, *j*). *T* = {*w*_*ij*_(*t*) | *t* = 1,2,3⋯} represents a set of weights on synapses(*i*, *j*) at different times.

According to the state of time *t* and *w*_*ij*_(*t*), the synaptic weight set *w*_*ij*_(*t* + 1) at time *t* + 1 can be obtained by *y*; pr(*σ*_*i*_) and po(*σ*_*i*_) represent the label set of presynaptic neurons and postsynaptic neurons of *σ*_*i*_, respectively.

If *σ*_*i*_ contains *b* pulses and *a*^*b*^ ∈ *L*(*E*), *E*/*a*^*c*^ → *αk*(*i*, *Q*) is used. If the rules in the system are excited, *c* pulses will be consumed. Then, the next step will be performed according to the value of *α*:

- For *α* = +, if 1 ≤ ∣*Q* − pr(*σ*_*i*_) | ≤*k*, *σ*_*i*_ selects all neuron tags in *Q* − pr(*σ*_*i*_) to create synapses. If ∣*Q* − pr(*σ*_*i*_) | >*k*, *σ*_*i*_ randomly selects *k* neuron tags in *Q* − pr(*σ*_*i*_) to create synapses. If *Q* − pr(*σ*_*i*_) = ∅ or pr(*σ*_*i*_) = ∅, *C* pulses are consumed but synapses are not established. In this case, the principle of synaptic creation rules is similar to that of standard rules of oblivion
- For *α* = −, if ∣pr(*σ*_*i*_) | ≤*k*, all synapses are deleted in pr(*σ*_*i*_). If ∣pr(*σ*_*i*_) | >*k*, *k* neurons are selected in pr(*σ*_*i*_) and the synaptic connection with each selected neuron is deleted
- For *α* = ∓, synapses are created at the time *t* and deleted at the time *t* + 1. Conversely, for *α* = ±, synapses are deleted at the time *t* and created at the time *t* + 1. In this case, the use of rules is similar to that of *α* = + and *α* = −. From time *t* to time *t* + 1, *σ*_*i*_ is always in an open state, but *σ*_*i*_ uses other rules at time *t* + 2

If *σ*_*i*_ has *k* pulses and *a*^*k*^ ∈ *L*(*E*), *k* ≥ *c*, the excited rule *E*/*a*^*c*^ → *a*^*p*^; *d* is used. When this rule is used, *σ*_*i*_ will delete *c* pulses. At the same time, *p* pulses are sent to all neurons connected to *σ*_*i*_ after *d* time intervals. When the excited rule is used to the *d*-th time intervals, *σ*_*i*_ is in a closed state. Rules and processing pulses can only be used by *σ*_*i*_ when the execution conditions are met. If *σ*_*i*_ uses the excitation rule *E*/*a*^*c*^ → *a*^*p*^ at *t*-th step, *σ*_*i*_ at *t*-th, *t* + 1 − th, ⋯*t* + *d* − 1 − th step is not executed. After *t* + *d* steps, *σ*_*i*_ is in the excited state.

If a neuron has *s* pulses, the rule of oblivion *E*′/*a*^s^ → *λ*, *s* ≥ 1 is used. When this rule is used, *σ*_*i*_ will consume *s* pulses. No new pulse will be produced.

The state of System P at a certain time is expressed as *C*_*r*_ = <*k*_1_/*t*_1_, ⋯, *k*_*m*_/*t*_*m*_ > , 1 ≤ *i* ≤ *m*, where *k*_*i*_ represents the number of pulses stored in neuron *σ*_*i*_ in this state; *t*_*i*_ represents the time taken for *σ*_*i*_ to be reactivated. At the beginning of System P calculation, all neurons meet the excitation rule conditions. By rules, the state of the system is transferred. *C*_1_⇒*C*_2_ means that the system is transferred from state *C*_1_ to state *C*_2_. When all neurons in the system have been activated, the termination state means that there are no rules in the neurons that can be activated again. If a system is able to calculate till the termination state, then the calculation is regarded as the one that can be terminated.

According to the state of time *t* and *w*_*ij*_(*t*), the synaptic weight set *w*_*ij*_(*t* + 1) is obtained at time *t* + 1. Theoretically, if there is a transfer of *M*_*t*_^*t*+1^ from time *t* to time *t* + 1 in the system, and the set of weights on the synapse is *w*_*ij*_(*t*). Then, under the transfer of *M*_*t*_^*t*+1^, the set function of synaptic weights at time *t* + 1 is *w*_*ij*_(*t* + 1) = *y*(*M*_*t*_^*t*+1^, *w*_*ij*_(*t*)).

### 2.4. Network Connection

Based on the above analysis, the boundary extraction image is combined with the neural network system of SN P systems. The parallelism of SN P systems and the flexibility of neural networks are taken full advantage.

**Figure 3:**
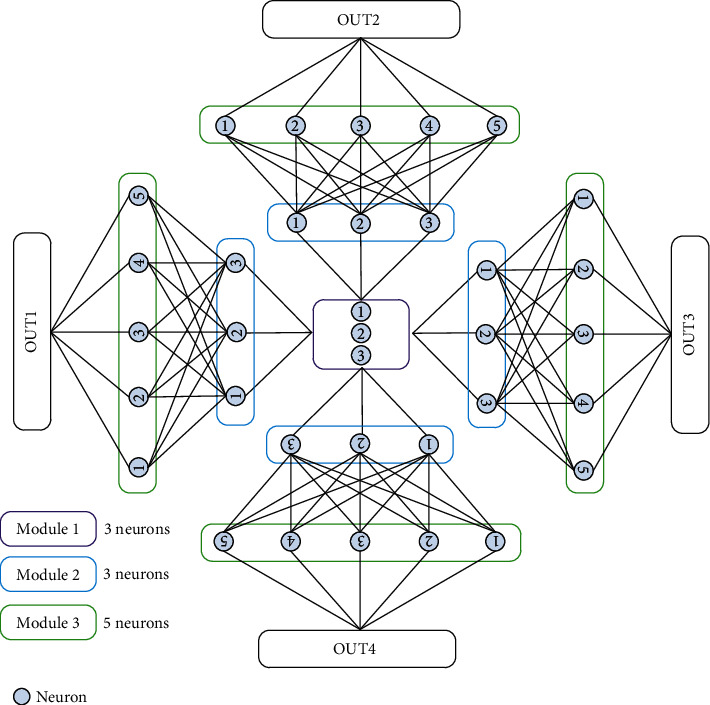
Network framework.

1. Mark the boundary of a pulmonary nodule as 1, which is regarded as a pulse signal. Nonboundary areas are marked as 0
2. Normalize the boundary image size of a pulmonary nodule to 5 × 7
3. The neurons are divided into three parts, as shown in Figure 3. The flow direction of a pulse signal is from Module 1 to Module 2 and then to Module 3. Three neurons of Module 1 establish the neural connection of Module 2 through the defined SN P systems rules, and the weights of all synapses are 1. There is only one excitation rule for the neurons of Module 2 and Module 3, that is, if the neuron contains pulses, the neuron is excited until the number of pulses in the neuron changes to 0, and the calculation is terminated. Module 2 has four layers, and each layer contains three neurons. Module 3 has four layers, and each layer contains five neurons. The neurons of Module 2 and Module 3 are connected by synapses

## 3. Experiment and Result Analysis

**Figure 4:**
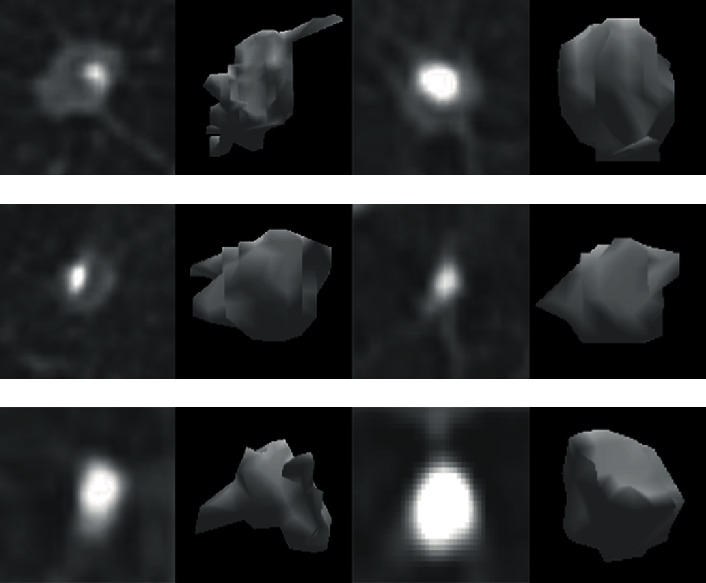
Experimental data.

All the experimental data are from the database of the International Early Lung Cancer Action Project and the American Association of Lung Imaging Databases, as shown in Figure 4. 514 pulmonary nodules with spiculation signs and 501 pulmonary nodules without spiculation signs are labeled by two professional doctors as the detection basis. The ratio of training data and test data is 1 : 1.

### 3.1. Image Segmentation

The area overlap measure (AOM) is used to evaluate the segmentation effect.

(13) $$\begin{array}{c} \text{AOM}\left(A,B\right)=\frac{S\left(A\cap B\right)}{S\left(A\cup B\right)}\times 100\%. \end{array}$$

AOM is the overlap degree of area. *A* is the standard image. *B* is the segmentation result image. *S* (.) represents the pixel number of the corresponding area. The larger the AOM value, the better the segmentation effect.

Different algorithms are used to segment common pulmonary nodules and pulmonary nodules with speculation sign, as shown in Table 1. It illustrates that the segmentation effect for common pulmonary nodules is better than that for pulmonary nodules with spiculation sign. That is because common pulmonary nodules have high gray value and high density, and pulmonary nodules with spiculation sign have high gray values including small protrusions. The fixed threshold [17] algorithm achieves segmentation of pulmonary nodules by selecting threshold artificially, and the result is good. But the threshold setting is manual. The gradient model [26] algorithm focuses on the boundary to extract pulmonary nodules. AAGSM [29] used an initial shape of pulmonary nodules to constrain segmentation of pulmonary nodules. LS [31] algorithm establishes the iterative model to achieve segmentation of pulmonary nodules. The Snake [38] algorithm establishes internal force and external force balance mechanism to extract pulmonary nodules. The Esnake [40] algorithm introduces the Otsu algorithm to improve Snake and achieves good results. On the basis of the Snake algorithm, our algorithm protects boundary information and suppresses falling into local minimum. It has a strong segmentation effect for common pulmonary nodules and pulmonary nodules with speculation sign.

### 3.2. The Speculation Discrimination Effect

**Figure 5:**
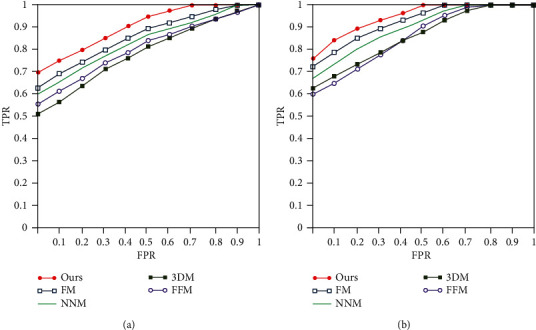
ROC curve. (a) The recognition results of original pulmonary nodule image by different algorithms. (b) The recognition results of original pulmonary nodule MIP image by different algorithms.

The ROC curve is introduced to measure the effect of all algorithms. The recognition results of the original pulmonary nodule image by different algorithms are shown in Figure 5(a), and the recognition results of different algorithms in MIP pulmonary nodule images are shown in Figure 5(b). It can be seen that the MIP algorithm can better reflect the boundary features of pulmonary nodules and improve the distinguishing effect of spiculation sign. The fractal model (FM) [34] uses the fractal operator to calculate the fractal degree of pulmonary nodules to judge the signs of pulmonary nodules. The nerve network model (NNM) [10] algorithm introduces a learning mechanism to realize feature learning, which requires a large number of samples to train parameters. 3DM [13] establishes a three-dimensional pulmonary nodule model and analyzes the pulmonary nodule signs from a spatial perspective, which can realize the identification of pulmonary nodule signs, but the algorithm has high complexity. The feature fusion model (FFM) [35] extracts the gray value and boundary information of pulmonary nodules to realize the identification of pulmonary nodules. Our algorithm fuses the pulmonary nodule information from three locations and proposes a time series analysis algorithm, which achieves good results. The proposed algorithm in this paper focuses on the boundary of the pulmonary nodule spiculation sign and integrates the SN P systems into the neural network. It gives full play to the advantages of the SN P systems and has a better effect.

## 4. Conclusion

In view of the recognition of pulmonary nodules with computer, a complete recognition system of speculation sign of pulmonary nodules is proposed from the doctors' perspective. The MIP algorithm is proposed to restore the three-dimensional local structure of pulmonary nodules. The improved Snake algorithm can extract the boundary information of pulmonary nodules completely. The neural network system based on SNP systems can help doctors to make accurate diagnosis with computer-aided. On the basis of existing datasets, we will expand the amount of data. By labeling the dataset, it is of great significance to integrate the imaging features and pathological features of different time periods into the model and carry out the research on the prediction of benign and malignant development trend of pulmonary nodules.

## Figures and Tables

**Table 1:** The effect comparison of algorithms.

Algorithm	AOM %	
	Common	Spiculation
Fixed threshold [17]	94	92
Gradient model [26]	85	76
AAGSM [29]	86	79
LS [31]	89	83
Snake [38]	91	84
Esnake [40]	93	87
Ours	94	90

## Data Availability

All used data is within the paper.
